# Editorial: Editors' showcase: frontiers in neuroanatomy

**DOI:** 10.3389/fnana.2025.1612905

**Published:** 2025-05-13

**Authors:** Javier DeFelipe, Joachim H. R. Lübke

**Affiliations:** ^1^Laboratorio Cajal de Circuitos Corticales, Centro de Tecnología Biomédica, Universidad Politécnica de Madrid, Pozuelo de Alarcón, Madrid, Spain; ^2^Institute of Neuroscience and Medicine INM-10, Research Centre Jülich GmbH, Jülich, Germany

**Keywords:** motor pathways, Cajal's lenticular tract, modular horizontal connections, alpha-synuclein and non-human primates, striatal interneurons and trans-neuronal labeling, secretagogin interneuron subtypes

The collection of articles presented here offers a compelling cross-section of contemporary neuroscience, illuminating fundamental aspects of neural circuitry and neurodegenerative models across species. Together, these studies underscore the diverse methodological approaches and conceptual insights that are reshaping our understanding of the brain.

Glover et al.'s work on hodological patterning in vertebrate motor circuits highlights how specific axonal trajectories underpin both the architecture and function of motor networks—a reminder of the intricate relationship between gene expression, neuronal positioning and connectivity. This theme of anatomical specificity is further explored in Puelles's study, which revisits Santiago Ramón y Cajal's observations. By leveraging modern tracing techniques and the Allen Mouse Brain Connectivity data, Puelles clarifies the trajectory of infrasubthalamic pyramidal tract collaterals, revealing novel insights into the organization of motor pathways and underscoring the value of revisiting classical neuroanatomical concepts with contemporary tools.

The complexity of cortical processing is elegantly addressed by Burkhalter et al., who delineate modular horizontal networks within the primary visual cortex. Their investigation into how local connections and feedback pathways converge in specialized cortical zones provides a nuanced perspective on the integration of visual information—a process fundamental to both perception and behavior. In parallel, Rico et al. extend the scope of neural research by developing a non-human primate model of disseminated synucleinopathy. This model not only opens new avenues for understanding the pathological spread of alpha-synuclein in conditions like Parkinson's disease with dementia and dementia with Lewy bodies but also lays the groundwork for more targeted preclinical drug screenings. Karube et al.'s innovative use of anterograde trans-neuronal labeling via AAV1 technology brings to light the complex interplay between dopaminergic neurons and striatal interneurons. The detailed mapping of these interactions deepens our insight into how dopaminergic signals are modulated at the single-cell level, a finding that carries significant implications for our understanding of motor control and its dysregulation in disease states. Finally, Tapía-González and DeFelipe present a refined neurochemical characterization of interneuron subtypes in the human frontal and temporal cortices through the study of secretagogin expression. By analyzing the coexpression of secretagogin with parvalbumin, calretinin, and neuronal nitric oxide synthase using triple immunostaining, they identify distinct patterns of colocalization that vary according to cortical area and layer. These findings underscore the regional neurochemical diversity of cortical interneurons and emphasize the need for further studies to comprehensively characterize the subtypes of secretagogin-expressing cells within the human cerebral cortex.

Collectively, these articles reinforce the notion that bridging traditional neuroanatomical frameworks with modern experimental techniques is key to unlocking the myriad mysteries of the brain. Whether by reassessing classical models or by pioneering innovative experimental approaches, these studies collectively chart a path toward a more integrated understanding of neural circuitry and brain pathology across species.

